# Outbreak investigation of Salmonella group D in staff and patients at Hamad Medical Corporation

**DOI:** 10.1186/1753-6561-5-S6-P93

**Published:** 2011-06-29

**Authors:** JA Alajmi, M Elsheikh, Badriya Al Ali, Fatema Al Habshi, Moza Elshaq, B Hosniyeh, C Joji, C Abraham, Bency Lukose, Bonnie George, Josephine C Ma, Leni Basco Garcia, Rida Alkhdor, J Ancy George William, CR Geogi

**Affiliations:** 1 Medicine Infection Control, HMC Qatar, Doha, Qatar; 2 Infection Control Quality Department, HMC Qatar, Doha, Qatar

## Introduction / objectives

I.C section was informed that around 17 staffs who had eaten dinner on Dec 6.2010 during Quality event and 27 cases of staff from different units and 7 cases from in patients areas had experienced gastrointestinal illness within 72 hr of finishing their meal .In total of 14 cases were stricken by this illne which subsequently identified as salmonella group D.

Objective:To investigate an increase in the number of Salmonella isolates detected to ascertain whether it was due to a nosocomial source, to identify the mechanisms of transmission, and to institute effective control measures to prevent future episodes.

## Methods

retrospective study was conducted among HCW to assess exposure to food served during outbreak andsubsequent illness. Exposed staffs were asked to complete Questionnaire .the Case definition was defined ,screening of staffs, food samples and surfaces were done.

## Results

The questionnaire was emailed to all 74 event participants, only 40 responded 17 staffs developed gastroenteritis. Stool cultures were available for 11 staffs. 7 staff became positive for Salmonella group D, and around 7 patients were reported to have Salmonella group D isolated in stool cultures. Screening of 130 staff from catering areas showed 6 catering staff were found to be positive for Salmonella group D, 2 of them returned from vacation and resumed their duties prior their clearance. Screening of different food items and surfaces were swabbed. All samples were negative for salmonella.

## Conclusion

This outbreak illustrates the potential for food handlers in health care setting who are infected with Salmonella of same serotype to be a source of transmission.Strict on Routine stool culture of food handlers, HCW education and proper hygienic practices are important to prevent such out break in the future.

## Disclosure of interest

None declared.

